# Radical Scavenging and Reducing Power of *Salvia mirzayanii* Subfractions

**DOI:** 10.3390/molecules13112804

**Published:** 2008-11-12

**Authors:** Mahmood Reza Moein, Soheila Moein, Saeid Ahmadizadeh

**Affiliations:** 1 Department of Pharmacognosy and Pharmaceutical Sciences Research center, Faculty of Pharmacy, Shiraz University of Medical Sciences, Karafarin St., Shiraz 71345-1583, Iran; E-mail: mrmoein@sums.ac.ir, mrezamoein@yahoo.com; 2 Biochemistry Department, Faculty of Medicine, and Infectious Disease Research Center, Hormozgan University of Medical Sciences, Shaheed Naser Blvd., Bandar Abbas 79149-64153, Iran.

**Keywords:** *Salvia mirzayanii*, Ethyl acetate subfractions, Antioxidant activity, Phenolic compounds, Flavonoids

## Abstract

In this research, the radical scavenging activity and reducing power of the ethyl acetate fraction and subfractions of *Salvia mirzayanii* (SM) have been investigated. The plant material was initially extracted with ethanol. The fractionation was carried out using liquid-liquid extraction, then the ethyl acetate fraction, which showed the greatest antioxidant activity, was selected. This fraction was submitted to column chromatography on a Sephadex LH 20 column eluted with pure MeOH to obtain subfractions A-G. No significant differences exist between the IC_50_ of *Salvia mirzayanii* ethyl acetate subfraction C (IC_50 = _37.9 ± 0.85), F (IC_50 = _40.05 ± 1.4) and quercetin (38.84 ± 0.86), (P > 0.05), indicating that the radical scavenging capacity of these two subfractions and quercetin (antioxidant standard) were similar. The reducing power of the ethyl acetate fraction was less than that of all subfractions, except for subfraction A. The greatest amount of phenolic compounds was found in subfraction E (55.23 ± 4.2) and the lowest in subfraction F (5.23 ± 0.18). The greatest total flavonoid content was established in subfraction D (1.84 ± 0.01) and the lowest was in subfraction A (0.108 ± 0.007).

## Introduction

It is well known that reactive oxygen species (ROS) such as ^•^O_2_ (superoxide anion), H_2_O_2_ (hydrogen peroxide), and ^•^OH (hydroxyl radical) are closely involved in various human diseases such as Alzheimer's disease, aging, cancer, inflammation, rheumatoid arthritis and atherosclerosis [1,2,3]. For several years, many researchers have been searching for powerful but non-toxic antioxidants from natural sources, especially edible or medicinal plants. Such natural antioxidants could prevent the formation of the above reactive species-related disorders in human beings without the use of synthetic compounds, which may be carcinogenic and harmful to the lungs and liver [4].

Also, antioxidants play an important role in nutritional by lengthening the shelf life of food and reducing nutritional losses and formation of harmful substances. However, the safety of synthetic antioxidants, such as butylated hydroxyanisole (BHA) and butylated hydroxytoluene (BHT) are now in doubted [5,6]. Thus, attention is now increasingly paid to the development and utilization of more effective and non-toxic antioxidants of natural origin. A great number of natural medicinal plants have been tested for their antioxidant activities and results have shown that the raw extracts or isolated pure compounds from them were more effective antioxidants *in vitro* than BHT or vitamin E [7,8,9], so, medicinal plants can be a potential source of natural antioxidants [10]. 

The high content of antioxidant polyphenolic compounds, such as catechin, ingested in the human diet represents an important source of non-nutritional antioxidants [11]. The benefits of certain non-nutrient antioxidants have been evaluated in several epidemiological studies [12]. In particular, it has been found that polyphenols exert protective effects against the development of cardiovascular diseases [13]. One of the plants containing polyphenols is *Salvia* which is one of the wide-spread members of the Labiatae (Lamiaceae) family. The Labiatae comprise about 900 herbs and shrubs, growing in the temperate and warmer zones of the world. Some of these species feature prominently in the pharmacopoeias of many countries throughout the world [14,15,16,17].

Previously, the ethyl acetate fraction of *Salvia mirzayanii* showed significant antioxidant activity in comparison with crude extract as well as the other fractions [18]. Antioxidant activity can be measured by radical scavenging activity and reducing power. Since several compounds are present in the ethyl acetate fraction based on its TLC (data not shown), the properties of *Salvia mirzayanii* (SM) subfractions were investigated. The total amount of phenolic and flavonoids compounds from the ethyl acetate subfractions has also been determined.

## Results and Discussion

The range of traditional applications of the *Salvia* herbs in domestic medicine seems to be endless: they have been used as a medication against perspiration and fever, as a carminative, spasmolytic, an antiseptic/bactericide, astringent, as gargles or mouthwashes against mouth, tongue and throat inflammation, as a wound healing agent, as a cure for skin and hair, against rheumatism and sexual debility, in treating mental and nervous conditions and as an insecticide [14,15,16,17]. Also, in addition to antioxidant and estrogenic activity, many *Salvia* species and their isolated constituents have demonstrated anti-inflammatory properties [19]. Sephadex LH-20 is routinely used for separation of flavonoids, including agylcones and glycones. It is a good stationary phase for the isolation of flavonoids from terpenoids. In this research, the ethyl actate subfractions, which were obtained using Sephadex LH-20 chromatography were investigated for antioxidant properties. The antioxidant activity of plant extracts can be measured by using reducing power and radical scavenging activity assays. 

**Figure 1:**
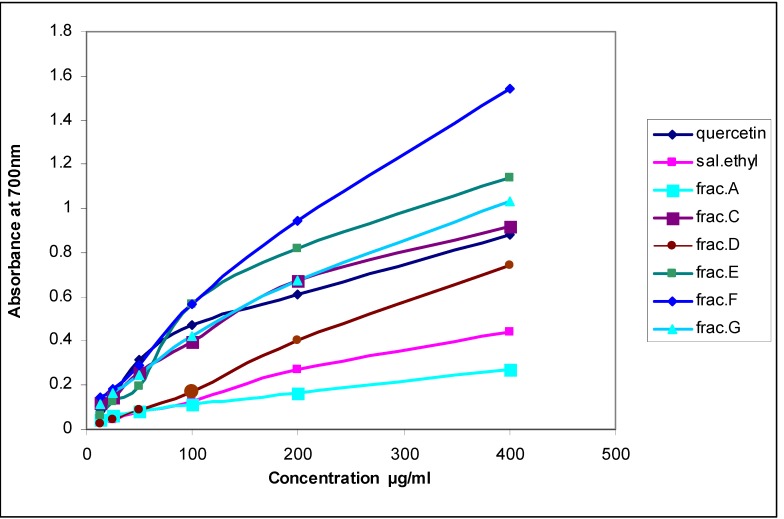
Reducing power of *Salvia mirzayanii* ethyl acetate subfractions compared with quercetin (was diluted 1:10) as standard. Subfraction C (was diluted 1:2) and subfraction D (was diluted 1:4).

In the reducing power assay, the more antioxidant compounds convert the oxidation form of iron (Fe^+3^) in ferric chloride to ferrous (Fe^+2^). The results of this research showed that the reducing power of the SM ethyl acetate fraction was less than all subfractions except for subfraction A (Figure 1, Table 1), meaning that during the fractionation process an increase in the antioxidant activity occurred.

**Table 1:** Concentration of *Salvia mirzayanii* subfractions at absorbance 0.5 compared with quercetin as standard in reducing power assay.

Samples	Concentration µg/mL (Absorbance 0.5)
Crude extract	ND
Sal. ethyl acetate fraction	169.04± 7.8
Sal. ethyl acetate subfraction A	> 400
Sal. ethyl acetate subfraction B	ND
Sal. ethyl acetate subfraction C	63.13± 2.8
Sal. ethyl acetate subfraction D	62.5 ± 3.04
Sal. ethyl acetate subfraction E	100± 5.2
Sal. ethyl acetate subfraction F	100± 4.8
Sal. ethyl acetate subfraction G	150± 5.4
Quercetin	10± 0.06

ND: non detected; Sal: *Salvia mirzayanii*

The reducing power of all ethyl acetate subfractions was less than quercetin (P < 0.001, Table 1) and this power of subfractions C and D were more than the other subfractions (P < 0.001, Table 1).

**Figure 2:**
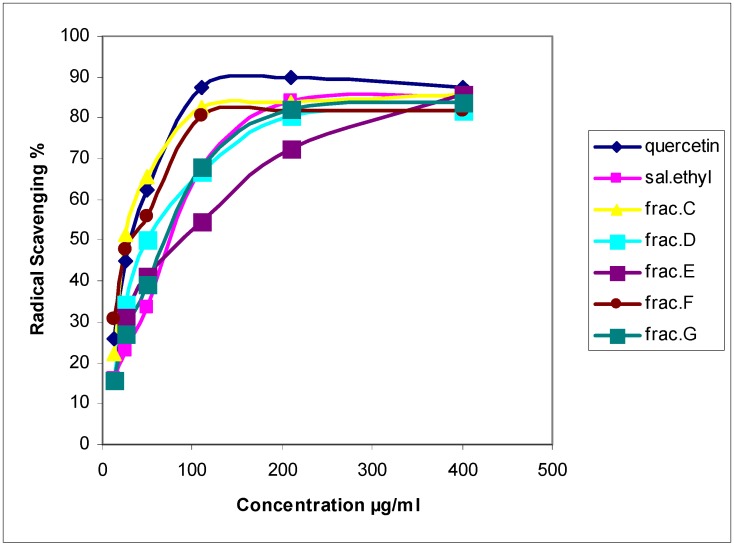
Radical scavenging activity of *Salvia mirzayanii* and sub-fractions compared with quercetin as a standard.

In the radical scavenging assay, when the DPPH is exposed to antioxidant compounds the purple color of DPPH changed to yellow. The more yellowish color of DPPH observed the greater the antioxidant activity of the compounds tested. The results of radical scavenging showed that, subfractions C and F possessed strong radical scavenging effect as far as quercetin (Table 2, Figure 2). It was observed a significant difference (P < 0.001) among the IC_50_ of two subfractions E, G and quercetin, which means that the radical scavenging activities of these two subfractions were less than quercetin. The results showed that subfraction C possessed highest radical scavenging and reducing power activities (Table1, Table 2).

The total phenolic content was determined by using the Folin-Ciocalteu method. The content of total flavonoids was also measured spectrophotometrically by using the aluminum chloride colorimetric assay.

**Table 2:** IC_50_ of *Salvia mirzayanii* subfractions compared with quercetin as standard.

Samples	IC_50 _µg/mL
Crude extract	ND
Sal. ethyl acetate fraction	63.2 ± 2.4
Sal. ethyl acetate subfraction A	NA
Sal. ethyl acetate subfraction B	ND
Sal. ethyl acetate subfraction C	37.9 ± 0.85
Sal. ethyl acetate subfraction D	62.5 ± 3.01
Sal. ethyl acetate subfraction E	84.2 ± 0.98
Sal. ethyl acetate subfraction F	40.05 ± 1.4
Sal. ethyl acetate subfraction G	68.1 ± 1.62
Quercetin	38.84 ± 0.86

ND: non detected; NA: non active; Sal: *Salvia mirzayanii*

In this study, a low correlation (R = 0.048) between phenolic compounds and flavonoids has been found; this correlation was also found by other researchers [20]. This could be explained by the presence of some chemical groups such as amino acids and proteins that can also react with Folin-Ciocalteu reagent [20]. The highest amount of phenolic compounds was in subfraction E (55.23 ± 4.2 mg/g, Table 3) and the lowest amount was in subfraction F (5.23 ± 0.18 mg/g). The highest amount of flavonoids was in subfraction D, (1.84 ± 0.01), and the lowest amount was in subfraction G (not detected). Moderate correlation (R = 0.65) was shown between the DPPH radical scavenging and total phenolic compounds. It has been suggested that compounds were likely to be contributing to the radical scavenging activity [20]. On the other hand, it may be possible that the radical scavenging activity of a sample can not predicted on the basis of its total phenolic content [20]. In our study, there was no a correlation (R= -0.172, Table 3) between plant flavonoid level and radical scavenging activity. Other authors have also found a low correlation between plant flavonoid levels and radical scavenging activity [20]. Finally, it seems that other types of natural antioxidants are present in the active subfractions as well as phenolic compounds. 

**Table 3:** The amount of total phenolics and flavonoids in *Salvia mirzayanii* subfractions.

Sample	Total phenolics content mg/g	Total flavonoid content mg/g
Sal. ethyl acetate fraction	49.23 ± 3.4	ND
Sal. ethyl acetate subfraction A	9.76 ± 0.7	0.108 ± 0.007
Sal. ethyl acetatesubfraction C	5.84 ± 0.15	1.28 ± 0.006
Sal. ethyl acetate subfraction D	14.36 ± 0.8	1.84 ± 0.01
Sal. ethyl acetate subfraction E	55.23 ± 4.2	0.958 ± 0.018
Sal. ethyl acetate subfraction F	5.23 ± 0.18	1.2 ± 0.006
Sal. ethyl acetate subfraction G	10.78 ± 0.9	ND

## Conclusions

Refractionation of an ethyl acetate fraction of *Salvia mirzayanii* by column chromatography on Sephadex LH-20 resulted in 7 fractions A-G which showed more antioxidant activities than the crude extract and the polar fractions. In radical scavenging activity, some of the subfractions (F and C) were as active as the standard (quercetin). In other words, by using column chromatography more active fractions can be eluted. According to the current results, we could not find any correlation between flavonoid content and radical scavenging effect. However, a moderate correlation was found between phenolic content and radical scavenging activity [20]. 

## Experimental

### General

DPPH (2,2-diphenyl-1-picrylhydrazyl radical), quercetin, gallic acid and Folin-Ciocalteau reagent were obtained from Sigma Chemical Co., St Louis, MO. All other reagents were obtained from Merck Chem.

### Extraction and fractionation of plant materials

Aerial parts of SM were collected before flowering in March 2004, from Fin (80 km from Bandar-e-Abbas, Capital of Hormozgan Province, Iran), and were identified and authenticated (voucher no.137) by Mr. M. Kamalinejad in the Depatrment of Pharmacognosy at Shahid Beheshti University of Medical Sciences, Tehran, Iran. The leaves were separated, dried at room temperature, and ground into powder (mesh< 35).

Aerial parts of SM (5 kg) were percolated with ethanol (31.8 L) for 1 week and the extract concentrated under vacuum at 40^○^C to give a crude extract (1.1 kg). A portion of the crude extract (452.6 g) was then suspended in 80% methanol (800 mL) and extracted petroleum ether (3×800 mL). The residue was extracted with chloroform (3×800 mL), and the concentrated MeOH fraction (271.2 g) was suspended in water. The fractionation was continued with ethyl acetate (3×500 mL) and followed by another fractionation with *n*-butanol (3×500 mL). Finally the ethyl acetate fraction (14.2 g) was loaded onto a Sephadex LH 20 column and eluted with pure MeOH to obtain subfractions A-G. 

### Measurement of Reducing Power

The reducing power of SM ethyl acetate, fraction and subfractions was determined using the method described previously [22]. A serial dilution of the extract was performed (200, 100, 50, 25 and 12.5 µg/mL) in 0.2 M phosphate buffer pH, 6.6 containing 1% ferrocyanate. The mixture was incubated at 50 ºC for 20 minutes. 10% trichloroacetic acid (TCA, 2.5 mL) was added to a portion of this mixture (5 mL) and centrifuged at 3,000 g for 10 minutes. The supernatant was separated and mixed with distilled water (2.5 mL) containing 1% ferric chloride (0.5 mL). The absorbance of this mixture was measured at 700 nm. The intensity in absorbance could be the measurement of antioxidant activity of the extract [22].

### Determination of antioxidant using DPPH

The antioxidant activity of SM ethyl acetate fractions, subfractions and the antioxidant standard were assessed on the basis of radical scavenging effect of the stable DPPH free radical. In a modified assay [21], a 100 mM solution of DPPH radical in methanol (200 µL) was mixed with an aliquot (20 μL) of fraction (or subfractions). The concentrations of fractions were 12.5-400 µg/mL. After mixing, they were left for 30 min at room temperature. The DPPH radical inhibition was measured at 490 nm by using a micro-plate reader model Stat Fax 2100, Awareness technology, Inc. The IC_50_ of each sample (concentration in µg/ml required to inhibit DPPH radical formation by 50%) has also been calculated. Tests were carried out in triplicate. The antioxidant activity (AOA) was given by:

100 – [(A) sample-(A) blank) × 100 / (A) control]

where "A" is the absorbance of the color formed in microplates wells. DPPH used as control, and blank contains methanol.

### Determination of total phenolic content

The content of total phenolic compounds in SM fraction and subfractions were determined by the Folin-Ciocalteu method [23]. For the calibration curve aliquots (1 mL) of 0.024, 0.075, 0.105 and 0.3 mg/mL of gallic acid methanol solutions were mixed with Folin-Ciocalteu reagent (5 mL, diluted ten –fold) and sodium carbonate (75 g/L, 4 mL). The absorption was read at 765 nm at 20 ^º^C after 30 min and the calibration curve was drawn. Methanol plant extract (10 g/L, 1 mL) was mixed with the same reagents as described above, and after 1 h the absorption was measured for the determination of plant phenolics. All determinations were performed in triplicate. Total content of phenolic compounds in plant methanol extracts in gallic acid equivalents (GAE) were calculated by the following formula:

C= c. V/m´

where C is the total content of phenolic compounds, mg/g plant extract, in GAE; c is the concentration of gallic acid established from the calibration curve, mg/mL; v is the volume of extract, mL; m is the weight of pure plant methanol extract, g.

### Determination of total flavonoid content

The total flavonoid content of SM subfractions was determined by using of a slightly modified colorimetric method described previously [24]. An aliquot of appropriately diluted sample solution (0.5 mL) was mixed with distilled water (2 mL) and subsequently with NaNO_2 _ 0.15 % solution. After 6 min, 4% NaOH solution (2 mL) was added to the mixture. Immediately, water was added to bring the final volume to 5 mL, the mixture was thoroughly mixed and allowed to stand for another 15 min. Absorbance of the mixture was determined at 510 nm versus a prepared water blank [25]. Quercetin was used as standard compound for the quantification of total flavonoid content. All values were expressed as milligram of quercetin equiv per 1 gram of extract. Data was recorded as mean ± SD for three replicates.

### Statistical analysis

IC_50_ values were calculated by linear regression. Means ± SD were calculated. The data were analyzed for statistical significance using one way ANOVA followed by Tukey post test. P values less than 0.05 were considered significant.
